# The Relationship between Organizational Justice and Quality Performance among Healthcare Workers: A Pilot Study

**DOI:** 10.1155/2014/757425

**Published:** 2014-04-03

**Authors:** Salwa Attia Mohamed

**Affiliations:** Department of Medical Surgical Nursing, Faculty of Nursing, Fayoum University, P.O. Box 63514, Fayoum, Egypt

## Abstract

Organization justice refers to the extent to which employees perceive workplace procedure, interactions, and outcomes to be fair in nature. So, this study aimed to investigate the relationship between organizational justice and quality performance among health care workers. The study was conducted at the Public Hospital in Fayoum, Egypt. The study included a convenience sample of 100 healthcare workers (60 nurses and 40 physicians) that were recruited. Tools used for data collection included (1) questionnaire sheet which is used to measure health workers' perception of organizational justices. It includes four types: distributive, procedural, interpersonal, and informational justice. (2) Quality performance questionnaire sheet: this tool was used to examine health workers' perception regarding their quality performance. It contained three types: information, value, and skill. The results revealed that a positive correlation was found between organizational justice components and quality performance among the various categories of health workers' perception (*P* ≤ 0.05). It has been recommended to replicate the study on a larger probability sample from different hospital settings to achieve more generalizable results and reinforce justice during organization of ministry centers in Egypt.

## 1. Introduction

Justice is a key issue for understanding organizational behavior [1]. During the past twenty-five years, the study of fairness has received major research attention from a variety of disciplines, including economics, psychology, law, and organizational science [2]. Cremer et al. [3] described organizational justice as “a dominating theme in organizational life.” The importance of studying organizational justice in the workplace has been underscored by findings that caused a sense of working for justice or lack of fairness in the workplace, which can lead to a decline in levels of organizational performance. Employees who perceive unfairness in the workplace may exhibit varying degrees of negative behavior. It can influence the employees' commitment to the organization and implementation of performance. Organizational change is any single action or set of actions that result in a shift in direction or process that affects the way the organization works. Change may affect the strategies an organization uses to carry out its mission, the processes for implementing strategies, the tasks and functions performed by the people in the organization, and the relationships between those people [4, 5].

Organizational justice is an umbrella term used to refer to individuals' perceptions about the fairness of decisions and decision-making processes within organizations and the influences of those perceptions on behavior [6]. Justice researchers have identified three or four specific types of justice, each referring to fairness perceptions in a specific set of work contexts: distributive justice, procedural justice, and interactional justice (sometimes broken down further into informational justice and interpersonal justice) [7].

Organizational justice research, which focuses on the role of fairness as a consideration in the workplace, has demonstrated that fair treatment has important effect on individual employee attitudes, such as satisfaction, absenteeism, and commitment [8]. In many studies, it has been suggested that fairness affects several employee attitudes and behaviors such as job satisfaction, organizational commitment, sabotage, turnover intention, stress, organizational citizenship behavior, job performance, and trust [9–12]. According to another description, it is the structure which affects the work attitudes of employees towards division of labor, wages, rewards, and recreation conditions along with determining the quality of social interaction [13]. Besides, it is emphasized that the perception of people about the rights and fairness in the organizational life is one of the definitions of organizational justice.

Organizational justice led to the rule of an atmosphere of trust and confidence, especially in institutions staffed by many professions and core professions aiding. organizational justice promotes an atmosphere of complacency among a team work one, is the health of the most important areas of social welfare, which guaranteed by the state for citizens and to hold the sector team medical work efficiently even lead services and promote high quality of care in work area [13].

According to Abdel Hamid [14] a statistically significant relationship among the level of the three dimensions of organizational justice (distributive and procedural transactions), the development of citizenship behavior among social workers in medical personnel, and the care of young people as a result of what they feel about fairness, equality, and self-strengthening exists.

Additionally, the impact of organizational justice perceptions on performance is believed to stem from equity theory. This would suggest that when people perceive in justice they seek to restore justice. One way that employees restore justice is by altering their level of performance. Procedural justice affects performance as a result of its impact on employee attitudes. Distributive justice affects performance when efficiency and productivity are involved [15]. Justice perceptions improve productivity and performance [16].

Bakhshi and Colleagues [17] showed a relationship among organizational justice, job satisfaction, and commitment. They also found a positive relationship among procedural justice, job satisfaction, and commitment. In a study of 507 hospital nurses, Brooks and Zeitz [18] found total quality management dimensions related to perceptions of procedural justice, and such perceptions mediated the effects of TQM on affective commitment.

According to Sirgy et al. [19], employees perceive the quality when their basic expectations of the work place and jobs are met appropriately. According to Maslow's hierarchy of needs, these expectations can be categorized from physiological needs (e.g., work place facilities) to self-actualization. Organizational justice is an important variable in how to meet these expectations and organizational needs. Research shows that perceptions of justice are strongly related to individuals' attitudes. Another research demonstrates that distributive justice affects attitudes about specific events (e.g., satisfaction with pay, satisfaction with one's performance appraisal), whereas procedural justice and interactional justice affect attitudes about the system (e.g., organizational commitment, trust in authorities) [20].

In recent years, healthcare industry has become a focus of research, especially in the context of hospital-based care. Among the healthcare professionals nurses appear to be the most discussed group in organizational justice literature [21, 22]. These shared justice perceptions, in turn, may create a situation that promotes or inhibits positive attitudes toward supervision and employee affective commitment to the organization [23].

### 1.1. Aim of This Study

The aim of the current study is to investigate the relationship between organizational justice and quality performance among healthcare workers.

### 1.2. Research Hypothesis

There is a significant relationship between organizational justice and quality of performance in health care workers.

## 2. Subjects and Methods

### 2.1. Design

A descriptive and correlation design was used in this study.

### 2.2. Setting

This study was carried out at all units of the Public Hospital in Fayoum, which is affiliated to the Ministry of Health in Egypt.

### 2.3. Sample

A purposive sampling technique was used to select the study sample of 60 nurses, and 40 physicians during the period from November 2013 to March 2013. The patients had been selected according to the following criteria: age ranged 18–60 years old (b) working in the selected setting for at least 6 months prior to the data collection to be oriented for working conditions, (c) able to express opinion about organizational justice and quality performance, and agree to participate in the study.

### 2.4. Tools

Data of this study was collected using the following tools.

#### 2.4.1. The Structured Interview Questionnaire (SIQ)

It was designed by the researchers based on the literature review and included sociodemographic data, namely, age, sex, marital status, and so forth.

#### 2.4.2. Organizational Justice Questionnaire Sheet

This tool was developed by Colquitt et al. [8] and Niehoff and Moorman [24] and was modified by the researcher to be applicable for measuring healthcare workers organization justice from his/her perspectives. It consists of 34 items that cover 3 types of justice, including distributive (13) items, procedural (11) items, and interpersonal (10) items. Participants used a 3-point likert scale that could be agree, almost, and disagree, which were, respectively, scored as 3, 2, and 1. Scoring system can be interpreted as higher scores reflect higher perceived amount of the type of fairness and lower scores reflect lower perceived amount of that type of fairness.

#### 2.4.3. Quality Performance Questionnaires Sheet

It was developed by researcher after review of the literature [25–27]. It includes thirty (30) items scale divided into three subscales, which are informational (11) items, value (10) items, and skill (9) items. Participants used a 3-point scale to report their perception of quality performance. Scoring system can be interpreted as higher scores reflect higher performance and lower scores reflect lower performance. It was tested for its reliability by the researcher using Cronbach's alpha test and the coefficient value was 0.81.

### 2.5. Ethical Consideration

Permission to conduct the study was obtained from the directors of the Public Hospital in Fayoum, which is affiliated with the Ministry of Health. Prior to the initial interview, the researchers introduced themselves to health care worker who met the inclusion criteria; each participant was fully informed with the purpose and nature of the study, and then an informed consent was obtained from participants who accepted to participate in the study. The researchers emphasized that participation in the study is entirely voluntary and participants can be withdrawn from the study and confidentiality was assured through coding the data.

### 2.6. Procedure

Official permission to conduct the study was obtained from the directors of hospital after explanation of the study purpose. Tool was developed by the researcher after reviewing the related literature. Content validity of the three tools was tested by 6 experts from social worker, 6 medical staff, and 3 nurse staff from Fayoum University, and the necessary modifications were done. Test-retest method was used to determine the reliability of the tool by applying this tool twice on 10 subjects who were excluded from the study. The reliability was 0.81. A pilot study was carried out on 10% of healthcare workers to assess the clarity of the statements and time required to complete the questionnaire. After giving consent, subjects completed the questionnaires, while they were in their work settings; completing the questionnaire took about 20–30 minutes. Data collection, review, and coding were completed during the period from November, 2013, to April, 2013.

### 2.7. Data Analysis

Statistical analysis was done using SPSS 12.0 statistical software packages. Data was presented in tables. Also for analysis of quantitative data mean and standard deviation were used. Spearman correlation analysis was used for assessment of the relationships among various scales. Statistical significance was considered at *P*  value ≤ 0.05.

## 3. Results

Table 1 revealed that the demographic characteristics of healthcare workers' sample were as follows: 85% of the participants were female; the proportion of female was higher among nurses (100%) than doctors (62.5%) (*P* > 0.05). Regarding age of participants, it was less 30 to 35 years for most of healthcare workers (*P* > 0.05). The highest nurses reported experience from five to nine years (38%), compared with doctors (37.5%) and nurses (38.33%) (*P* > 0.05).

Table 2 shows that that the difference among doctors, and nurses mean scores of organizational justice as regard to distribution, interpersonal, and quality performance as regard to cognitive and skill (65.2 25.4, 61.7 24.0; 65.8 19.7, 61.1 18.3; 62.8 20.7, 72.9 24.8; 60.2 16.2,68.2 18.4, resp.). The results also show that highly statistically significant differences between mean scores of the organizational justice and quality performance as regard procedural and value for the study groups (*P* < 0.01).

Table 3 presents the relation between organization justice components and quality performance among healthcare workers. There is a positive statistically significant correlation between organization justice components and quality performance (*P* < 0.01).

## 4. Discussion

Justice is a key issue for understanding organizational behavior [1]. Increasing attention has been paid in recent years to the issue of organizational justice and its impacts on organizational outcomes. The present study aims at investigating the relationship between organizational justice and quality performance. In the meantime, there is a relationship among distributive justice, procedural justice, interactional justice, and quality of performance.

Nurses in the present study perceived interpersonal justice as the highest organizational justice. This finding may be due to the result that many nurses considered their superior treating them with less dignity and respect by supervisors. This supported by Colquitt et al. [28]. Interpersonal justice reflects the degree to which people are treated with politeness, dignity, and respect by authorities. The experience of interpersonal justice can alter reactions to decision outcomes, because sensitivity can make people feel better about an unfavorable outcome. Interpersonal treatment includes interpersonal communication, truthfulness, respect, propriety of questions, justification, honesty, courtesy, timely feedback, and respect for rights. In a recent study, Tzafrir and Gur [29] found out that higher levels of trust in managers impact the way the employee perceives the quality service in the organization and the service he gives to customers.

The study also shows that nurses perceived procedural justice as the lowest organizational justice. This means that procedural justice towards employees is a basis for employee commitment [30]. Procedural justice influences individuals' perceptions of fairness with regard to pay raises and promotions as well as organizational commitment and job satisfaction. In contrast to study by Zakaria and Gheith [31] who stated that the nurses at King Abdullah Hospital perceived justice distribution as the highest organizational justice, Lambert [32] found that justice distributive was the lowest, whereas interpersonal justice was the highest. In addition, Cropanzano et al. [33] suggested that employees not only consider the different types of justice (i.e., distributive, procedural, and interactional) but also consider the agent of the situation that is perceived as fair or unfair. In a sense, perceptions of distributive justice are based on the exchange principle: employees evaluate the organizational outputs they receive compared with their inputs to determine whether it is a fair outcome [32]. Distributive justice deals with perceptions of the ends and procedural justice deals with perceptions of the means. In agreement Lambert et al. [34] found that distributive justice and procedural justice are associated with increased job satisfaction and organizational commitment among correctional staff.

The results of the present study revealed that there were statistically significant positive correlation between nurses' perception of organizational justice and quality performance components. The higher levels of organizational justice, particularly procedural justice, perceptions are related to more positive quality performance. This is in line with Posthuma et al. [35] who stated that justice employee participation and control in decision-making processes through managers should allow employees to provide information to the decision maker before a decision is made leading to quality service. Beecroft et al. [36] also stated that allowing nurses to participate in decision-making and providing them autonomous and empowered behavior, communication, collaboration, and openness in relation with other employees increased job satisfaction, improved the quality of care, and facilitated the recruitment and retention. This means that organizational procedural justice provides employees with indirect influence over the outcome of the decision-making process by means of process control. Process control also allows employee's possibility to express his/her view during the decision-making process [37].

Our study results also revealed that there were statistically significant positive correlation between health worker perception of organizational justice and quality performance components. The higher levels of organizational justice, particularly procedural justice, perceptions are related to more positive quality performance, particularly, informational justice. This was found to be in line with study by Tangirala and Ramanujam [38] who investigated the cross-level effects of procedural justice on employee's silence by surveying of sample size of 606 nurses divided in 30 workgroups. The research concluded that the procedural justice environment moderated the effects of employee's silence and the effects of employee silence were less than those where procedural justice environment does not exist. In contrast, Zakria et al. [31] interpreted the relationship between nurses' perception of organizational justice and their organizational commitment at King Abdullah Hospital. They found that the higher levels of organizational justice, particularly distributive justice, perceptions are related to more positive correlation with organizational commitment.

Findings of the present study are almost compatible with findings of the previous researches. Meta-analytic reviews have yielded a moderately strong positive relationship among procedural justice, the perceived fairness of decision-making processes, and task performance. That relationship suggests that taking steps to make decision-making more fair may actually improve individuals' fulfillment of task duties [39]. Ambrose et al. [40] argued that distributive justice affects attitudes about specific events (e.g., satisfaction with pay, satisfaction with one's performance appraisal), whereas procedural justice and interactional justice affect attitudes about the system (e.g., organizational commitment, trust in authorities). As a result, employees will be more likely to feel satisfied and subsequently perform their duties as specified in their job descriptions. Some studies [41, 42] support the positive relationship between procedural justice judgments and task performance. de Cremer [43] supported this notion by saying that if unfair procedures are used trust will be low and employees will be most likely to show low commitment.

The data of this study revealed that the organizational justice has more positive relationship with quality performance, particularly informational justice. Results are consistent with Aryee et al. [44] who found an equitable exchange relationship between managers and employees motivating employees to act in accordance to organizational norms that emphasize service quality. This attributed by employees who are satisfied with the justice system in the organization; they are more committed to delivering quality service to clients [45]. The global health worker view on the organization justice, working conditions, and relationship between managers and coworkers impacts quality performance.

## 5. Conclusion and Recommendation

The study findings point out that there are deficiencies in organizational justice in the medical agency, this shortcoming among the members of the medical team most likely to get a high percentage of the organizational justice dimensions (distributive, procedural, transactions) to doctors and due to the rule of general culture, and give priority attention to doctor as the most important in the health system. Also, quality performance components such as skill side and moral/value side higher rate in nurses than physician.

Consequently, quality performance will be available only to the availability of organizational justice, which causes them to develop and improve their quality professional performance.

### 5.1. Recommendations

Based on the result of the present study, the following recommendations are made:

1. decisions must be based on information that is accurate and participation of members of the medical team in decisions related to their work;
2. hospital managers should openly describe the fair procedures they are using and explain decisions thoroughly in a manner demonstrating dignity and respect using unbiased and accurate information;
3. providing job information about hospital policies, rules, and regulations;
4. leadership should focus on creating positive emotional atmosphere by setting aside time for regular confidence building sessions and providing rewards for achievements;
5. future studies should go beyond this to assess the possible reasons and effect of the relationship between organizational justice and quality performance. This study also suggests more research is needed to examine the relationship between organizational justice and other variables, such as organizational citizenship behavior and commitment.

## Figures and Tables

**Table 1:** Demographic characteristics of health workers.

Items	Total (*n* = 100)	Doctors (*n* = 40)	Nurses (*n* = 60)	*P* value
	*N* (%)	*N* (%)	*N* (%)	
Sex:				
Female	85 (85)	25 (62.5)	60 (100)	*P* < 0.05
Male	15 (15)	15 (37.5)	0 (0)	
Age in years:				
<30	29 (29)	9 (22.5)	20 (33.3)	*P* < 0.05
30–35	27 (27)	12 (30)	15 (25)	
35–40	16 (16)	8 (20)	8 (13.3)	
40–45	19 (19)	7 (17.5)	12 (20)	
45–50	7 (7)	2 (5.5)	5 (8.33)	
>50	2 (1.8)	2 (5.5)	0 (0)	
Experience:				
<5 years	20 (20)	9 (22.5)	15 (25)	*P* < 0.05
5–9	38 (38)	15 (37.5)	23 (38.33)	
10–14	25 (25)	10 (25)	15 (25)	
15+	17 (17)	9 (22.5)	8 (13.33)	

**Table 2:** The mean and SD of the study sample's perception of organizational justices and quality performance among healthcare workers (*n* = 100).

Items	Doctors = 40	Nurses = 60	*P* value
	Mean ± SD	Mean ± SD	
Organizational justice			
Distributive justice	66.7 ± 25.4	60.3 ± 24.0	*P* < 0.05*
Procedural justice	65.6 ± 21.6	55.5 ± 18.1	*P* < 0.01
Interpersonal justice	65.8 ± 19.7	61.1 ± 18.3	*P* > 0.05
Quality performance			
Cognitive	62.8 ± 20.7	72.9 ± 24.8	*P* < 0.05
Value/moral	73.8 ± 22.1	83.8 ± 25.1	*P* < 0.01**
Skill	60.2 ± 16.2	68.2 ± 18.4	*P* < 0.05

**P value is significant at the 0.05 level; *P value is highly significant at the 0.01 level.

**Table 3:** The relation between organization justice and quality performance among healthcare workers categories (*n* = 100).

Items	Independent variables	Doctors		Nurses	
Dependent variables		*r*	*P* value	*r*	*P* value
Distributive justice	Informational	0.87	<0.01	0.76	<0.01
	Value	0.75	<0.01	0.62	<0.01
	Skill	0.84	<0.01	0.68	<0.01

Procedural justice	Informational	0.72	<0.01	0.84	<0.01
	Value	0.61	<0.01	0.73	<0.01
	Skill	0.70	<0.01	0.62	<0.01

Interpersonal justice	Informational	0.65	<0.01	0.79	<0.01
	Value	0.72	<0.01	0.76	<0.01
	Skill	0.78	<0.01	0.71	<0.01

Total organizational justice	Informational	0.83	<0.01	0.69	<0.01
	Value	0.68	<0.01	0.73	<0.01
	Skill	0.72	<0.01	0.75	<0.01
